# Review: β-glucans as Effective Antibiotic Alternatives in Poultry

**DOI:** 10.3390/molecules26123560

**Published:** 2021-06-10

**Authors:** Betty Schwartz, Vaclav Vetvicka

**Affiliations:** 1 Institute of Biochemistry, Food Science and Nutrition, The School of Nutritional Sciences, The Robert H. Smith Faculty of Agriculture, Food and Environment, The Hebrew University of Jerusalem, Rehovot 761001, Israel; 2 Department of Pathology, University of Louisville, Louisville, KY 40202, USA; vaclav.vetvicka@louisville.edu

**Keywords:** β-glucans, poultry, immunomodulatory, antibiotics

## Abstract

The occurrence of microbial challenges in commercial poultry farming causes significant economic losses. Antibiotics have been used to control diseases involving bacterial infection in poultry. As the incidence of antibiotic resistance turns out to be a serious problem, there is increased pressure on producers to reduce antibiotic use. With the reduced availability of antibiotics, poultry producers are looking for feed additives to stimulate the immune system of the chicken to resist microbial infection. Some β-glucans have been shown to improve gut health, to increase the flow of new immunocytes, increase macrophage function, stimulate phagocytosis, affect intestinal morphology, enhance goblet cell number and mucin-2 production, induce the increased expression of intestinal tight-junctions, and function as effective anti-inflammatory immunomodulators in poultry. As a result, β-glucans may provide a new tool for producers trying to reduce or eliminate the use of antibiotics in fowl diets. The specific activity of each β-glucan subtype still needs to be investigated. Upon knowledge, optimal β-glucan mixtures may be implemented in order to obtain optimal growth performance, exert anti-inflammatory and immunomodulatory activity, and optimized intestinal morphology and histology responses in poultry. This review provides an extensive overview of the current use of β glucans as additives and putative use as antibiotic alternative in poultry.

## 1. The Poultry Immune System 

The avian immune system strongly resembles defense reactions of all mammals and can be divided into two different types: innate and adaptive. Innate immunity is the most basic system birds use to fight off foreign antigens. Adaptive immunity is the more advanced system utilized when the innate immune system fails to protect against invading pathogens.

Since chickens lack encapsulated lymph nodes, they have extensive networks of mucosa-associated lymphoid tissues (MALT) such as the gut-associated lymphoid tissues (GALT) that line the intestinal tract and are important for normal immune system functioning. The GALT contains the cecal tonsils (CT), Peyer’s patches (PP), the bursa of Fabricius, and Meckel’s diverticulum. These structures are well defined prior to hatching; however, further maturation takes place as antigens are introduced into the structures [1]. The CT is the largest tissue in the GALT and is home to both T and B-lymphocytes. Because it contains both types, it plays a vital role in both antibody production and cell-mediated immune responses. The Meckel’s diverticulum, located on the small intestine, contains germinal centers, where mature B cells proliferate and differentiate into plasma cells producing different classes of antibodies [2].

Bacterial infections and parasitism of the intestinal tract in poultry are an important concern for both animal health and productivity. The use of antibiotics has reduced bacterial infections to some extent, but is problematic due to the development of antibiotic resistance. This problem limited or even prohibited the use of antibiotics for prophylactic reasons and resulted in several antibiotics being eliminated from the toolbox available to poultry producers. Overuse of antibiotics use plays a major role in the emerging public health crisis of antibiotic resistance [3].

## 2. Bacterial Infections and Parasitism of the Intestinal Tract in Poultry

The most common challenge of the intestinal tract of poultry is to struggle with bacterial infections. Infections from *Salmonella typhimurium*, *Escherichia coli,* and *Clostridium perfringens* are roughly some of the most usual pathogenic bacteria associated with poultry production. *Salmonella typhimurium* are Gram-negative, facultative anaerobic, non-spore forming bacteria belonging to the *Enterobacteriaceae* family, mostly found in the gastrointestinal tract of many mammals and specially poultry [4]. Fecal shedding allows *Salmonella* to be transmitted among birds and thus *Salmonella* spp. are so widespread in poultry production. It was widely demonstrated that *Salmonella* spp. infection is the etiological agent responsible for salmonellosis in both humans and animals [5]. *Escherichia coli* is a Gram-negative bacterium that has been known for ages to easily and frequently exchange genetic information through horizontal gene transfer with other related bacteria [6]. *E. coli* is a commensal organism living in the intestines of both humans and animals. However, some strains have been reported to cause gastrointestinal illnesses [7]. *Clostridium* is a genus of Gram-positive obligate anaerobic bacteria, which includes several significant human pathogens. Spores of *Clostridium* normally inhabit soil and intestinal tract of animals and humans [8,9]. C. perfringens is known to cause necrotic enteritis in poultry [10]. 

As alluded to earlier, bacterial infections alter the integrity of the gastrointestinal tract (GIT) and even may induce its significant destruction. Similarly, parasites of the genus *Eimeria* are organisms which invade the avian GIT and induce its alteration causing coccidiosis, an enteric disease of major economic importance worldwide. *Eimeria* species are obligate intracellular parasites that exhibit a complex life cycle with developmental stages alternating between the external environment and intracellular phase within the host [11]. Pathogenic *Eimeria* species belong to the phylum Apicomplexa, in particular *Eimeria maxima*, *E. tenella*, and *E. acervulina* [12]. Currently, seven species of *Eimeria* are known to infect chickens and differ in pathogenicity and variations between these *Eimeria* species include extent of pathogenicity and the ability to colonize specific sites of the intestine. *Eimera* infection burdens huge economic losses annually in avian farming. In addition to mortality that may arise from these parasites, a significant economic loss from morbidity as a result of a reduction in feed intake, nutrient, and energy digestibility, and performance; the destruction of the villi and crypt (shorter and thicker villi), and a reduction in tight junction functionality have been reported [11]. One of the outcomes of *Eimeria* spp. infection is that it usually facilitates secondary infections such as necrotic enteritis caused by *Clostridium perfringens*.

The severity of bacteria or protozoan disease will depend on several factors such as the load of the pathogen to which the bird is exposed to or the age of the chicken and others. Cumulatively, bacteria or protozoan infection results in a high number of sick birds, producing high mortality due to severe necrotic enteritis resulting from these diseases, conditions that ultimately could lead to significant economic losses.

## 3. Antibiotic Use in Poultry Production and Its Effects on Bacterial Resistance 

Bacterial infections in poultry provide a very important component of farming that affects both animal welfare, health, and animal productivity. A surge in the development and spread of antibiotic resistance has become a major cause for concern. Antibiotic resistance (AR) is defined as the ability of bacteria to resist the killing effects towards an antibiotic treatment that was previously demonstrated to be susceptible [13]. The rapid rise in the development and spread of AR is the main cause for concern and thus represents an issue of global interest [14].

The use of low concentrations of antibiotics, also referred to as subtherapeutic (STA) treatment, are given as additives in feed and/or water with the aim to improve daily weight gain and feed efficiency through alterations in disease suppression [15]. STA has reduced the impact of bacterial infections, but has led to concerns regarding development of antibiotic resistance. Over the past few decades, no major new types of antibiotics have been produced and almost all known antibiotics are increasingly losing their activity against pathogenic microorganisms, especially those used in poultry farming [16]. The antibiotic resistance crisis has been attributed to the overuse and misuse of these medications, as well as a lack of new drug development by the pharmaceutical industry due to reduced economic incentives and challenging regulatory requirements [17]. The use of antimicrobial agents in animal husbandry has been linked to the development and spread of resistant bacteria. It is known that worldwide, more than 60% of all antibiotics that are produced find their use in animal production for both therapeutic and non-therapeutic purposes [18].

Infections due to bacteria in poultry implies an important concern for both the bird’s health and efficient production. For example, *Salmonella* and *Campylobacter* bacteria are extremely dangerous food borne pathogens [19]. As mentioned above, the subtherapeutic use of antibiotics has reduced the impact of these and other bacterial infections, but concomitantly raised concerns about the development of antibiotic resistance [17]. This situation has caused the withdrawal of several antibiotics from the toolbox available to poultry producers. As poultry producers work to reduce the use of antimicrobials, alternative management strategies are being developed. Thus, there has been increased interest in ‘natural’ feed additives that can stimulate the immune system of poultry. Poultry meat products are among the most consumed products worldwide. Since many essential antibiotics are employed during poultry production in numerous countries, it may contribute to threatening the population wellbeing of using such antimicrobial products and thus causing the development of microbial resistance in poultry settings [15]. The levels of multi-drug resistant bacteria have also increased. Therefore, the need for alternative management strategies is bigger than ever [20]. 

As poultry producers work to reduce the use of antimicrobials, alternative management strategies need to be developed. There has been some research to show that β-glucans may play a role in replacing antibiotics and stimulating the immune system. 

## 4. Beta-glucans

β-glucans are carbohydrates made of complex glucose polymers that provide the major structure found in the cell wall of yeast, fungi, algae, and cereal grains such as oats and barley [21]. The structures of β-glucans vary depending on the original source and the type of linkages present on the glucose polymers [22]. β-glucans consist of a backbone of glucose molecules linked at the 1 and 3 carbon atoms. The six-sided glucose rings are connected together in linear or branched forms with glycosidic linkages. The structure of these glycosidic linkages will affect the functionality of the β-glucan molecules. When comparing fungal and yeast glucans to barley β-glucans, fungal and yeast β-glucans have side-chains linked at the 1 and 6 carbon atoms. In contrast, barley β-glucans have the glucose molecules linked at the 1 and 4 carbon atoms with some linkages between the 1 and 3 carbon atoms. When comparing fungal and yeast β-glucans, fungal β-glucans typically have shorter side chains than those found in yeast and this gives them a branched structure. β-glucans are polymers of glucose extracted from yeast cell walls, bacteria, fungi, and cereals such as oats and barley [23]. β-glucans from yeast and fungi consist of a backbone of glycopyranosyl molecules joined by 1,3-β-links; from this backbone, side chains can be joined by 1,6-β-links, producing a branched molecular structure. Fungal β-glucans have short branches while they are long in yeast. Cereal cell wall consists of not-branched β-glucans with glucopyranose molecules linked by 1,3-β and 1,4-β linkages. Barley and most cereal grains contain unbranched β-glucan chains [24]. Until recently, biologically efficient β-glucans were supposed to have similar structures: a main chain of β-(1→3) bound d-glucopyranose molecules (for better perspective, imagine beads on the string) to which some d-glucopyranoses are randomly connected by β-(1→6) linkages, causing a different degree of branching in different glucans. The detailed structure of β-glucans from dissimilar sources differs as well as their biological activity. In native β-glucans, their fibrils are composed from organized parts in which the main chain is coiled to a triple helix. These regions are combined with single or double filaments of β-(1→3)-d-glucopyranoses. The triple helix, formed by three hydrogen bonds and stabilized by side chains, is probably present only in high-molecular β-glucans with molecular weight over 90 kDa [25]. Diverse data on comparison of structure, molecular size, and biological effects can be found in the literature. However, despite extensive research, we cannot say which physicochemical characteristics will guarantee a highly active β-glucan and without substantial biological testing, we cannot even guess.

## 5. Beta-glucans and the Poultry Gastrointestinal and Immune System

There has been increased interest in natural feed additives that can stimulate the immune system of poultry. There has been some research showing that β-glucans may play a role in replacing antibiotics and stimulating the immune system [26]. It was reported that β-glucan was effective in promoting growth of broiler chickens and improving meat quality [26,27,28]. 

Feeds containing no chemical additives are increasingly used in poultry nutrition. Since antibiotic growth promoters have been discredited by consumer associations, there has been an increasing trend in formulating diets without supplementation of synthetic drugs and chemical additives and many scientists are dedicated in finding natural alternatives to replace antibiotics as growth promoters [29]. 

The use of β-glucans is generally more widely accepted by consumers than antibiotics even at low dose [28]. β-glucans from the yeast cell wall and mushrooms have been shown to stimulate both specific and non-specific immune responses and improve the chicken growth performance [21,30] and quality of meat [31]. β-glucans derived from yeast and fungi, so-called β-(1→3)(1→6)-β-glucans, have been shown to exert beneficial effects when administered as supplements to poultry [32]. 

Dietary supplementation with yeast β-glucans to broiler chickens has been shown to increase the macrophage phagocytic activity [33,34], indicating that yeast β-glucans may play an important role in the activation of both innate and adaptive immune systems in these animals. They activate macrophages, a key component of the non-specific immune system. Additionally, yeast β-glucan supplementation of the broiler chicken diet also results in larger lymphoid organs [33], the organs that produce lymphocytes. Lymphocytes are the common precursor cells for both the adaptive immune system as well as for natural killer cells, involved in defending the host against virus infections. β-glucans derived from the yeast *Saccharomyces cerevisiae* have been demonstrated to exert the most significant positive effect [35]. An increasing body of evidence demonstrates health benefits of β-glucans in poultry [36]. The newest studies suggested that β-glucan supplementation might reduce or even completely replace antibiotics in chicken [37]. 

Other studies found amelioration of immunosuppression [38] and increased efficacy of vaccines [39]. The exact mechanisms for these effects are not clear. Among possible explanations might be stress reduction, effects on gastrointestinal tract microbiota [40] on gut permeability [41], and reduction of the negative effects of lypopolysacharide on feed intake. However, these effects will depend on the type of glucan used, since barley-derived β-glucans have negative effects on the digestive tract of chicken resulting from increased intestinal viscosity [42]. In contrast, β-glucans derived from cereals, characterized by a different structural composition, (β (1→3)(1→4)-β-glucans), exert a serious harmful effect on either poultry health or performance [42]. 

The immunomodulatory effects of β-glucans in mammals have been extensively well documented. Even though that there are several similarities between mammalian and avian immune systems, there are still differences that not allow to extrapolate mammal immune research to poultry immune research.

A number of studies conducted hitherto have demonstrated that β-glucans may play a role in replacing antibiotics and stimulating the immune system. Several studies involving administration of β-glucans have been demonstrated to improve the gut health of poultry in general or those suffering from infection with pathologic bacteria [43]. β-glucans have been demonstrated (i) to increase the flow of new immunocytes into the various lymphoid organs, (ii) to upregulate the function of macrophages, (iii) and to afford an effective anti-inflammatory immunomodulator agent [36]. Therefore, β-glucans may provide a tool for producers trying to reduce or eliminate the use of antibiotics in poultry diets.

β-glucans have been shown to stimulate the proliferation of white blood cells, which in turn stimulates the immune system. The β-glucans are transported to the small intestine, then passed through the Peyer’s patches in the GALT, and subsequently moved around the body [36]. The presence of β-glucan is followed by its binding to specific receptors such as CR3 and Dectin-1 and results in stimulation of macrophages, higher formation and secretion of antibodies, and increasing activity of natural killer cells. In addition to direct stimulation of both specific and non-specific immunity, β-glucan can also influence the expression of immune-related genes and proteins. The stimulation in immune functioning serves to combat the negative effects of enteric infection or immune suppression due to high stress rearing conditions [36]. 

In addition to its role in nutrient digestion and absorption, the lining of the digestive tract acts as an effective barrier against pathogen invasion. The lining of the digestive tract provides the innate defense barrier against most intestinal pathogens. The integrity of the digestive system is maintained by the family of proteins denominated tight junctions (TJ), which include occludin, claudin, and junctional adhesion molecules [44]. Claudin and occludin protein families thus maintain intestinal integrity to form a solid barrier from pathogen invasion [45]. Intimate contact of pathogen bacteria to the intestinal cells is essential for intestinal barrier alteration to occur [46].

The goblet cells of the gastrointestinal tract are specialized cells able to produce and secrete mucus. Goblet cells in the intestinal mucosa secrete mucus, which provides the first line of defense against intestinal injury. The role of mucins is to attract microbe adhesion in order to increase elimination of microbes, and thus prevent physical and chemical injury by pathogens [47]. When the mucus layer is disturbed, adhesion of microbes to the intestinal epithelial surface may increase epithelial permeability and reduce the absorption of nutrients. 

It has been shown that β-glucans stimulate the production and flow of new immune cells into the various lymphoid organs, increasing the protection from potential invaders [36]. Following a proper immune response such as exposure to β-glucans, macrophages recognize specific markers on the surface of the pathogens, the Microbial Associated Marker Proteins (MAMPs). MAMPs subsequently bind to the receptors on the phagocytic cells known as Toll-like Receptors (TLRs) [48]. Birds have 10 known TLRs [49], being the key TLRs in chickens TLR-2 that recognizes peptidoglycan, TLR- 4 that binds LPS common in gram-negative bacteria, TLR-5 that recognizes flagellin common in bacteria flagella, and TLR-21 which recognizes unmethylated CpG DNA commonly found in bacteria [49]. 

Macrophages isolated from β-glucans-challenged chicks are able to bind MAMPs of the invading pathogens to the TLRs on their outer surface and bring the organism into their cytoplasm in the form of a phagosome. The phagosome then fuses with a lysosome and the enzymes in the lysosome degrade the engulfed organism. Therefore, it is legitimate to claim that macrophage function is responsive to dietary β-glucans, and dietary supplementation with β-glucans from the yeast *Saccharomyces cerevisiae* has been shown to increase phagocytic activity in broiler chicks [50]. Similarly, dietary supplementation of β-1,3-1,6-glucan derived from the fungus *Schizophyllum commune* was demonstrated to increase the phagocytic and bactericidal capabilities of intestinal macrophages [51]. 

Macrophages express and produce the enzyme inducible nitric oxide synthase (iNOS). This enzyme results in the production of nitric oxide, which reacts with superoxide anions to generate toxic derivatives that allow the macrophage to kill several types of pathogens. Cox et al. [52] reported that β-glucan supplementation upregulated iNOS intestinal expression, implying the increased capacity of intestinal macrophages to destroy pathogens. Avian macrophages also produce IL-8 (referred to as CXCLi2). IL-8 is a chemokine demonstrated to be an important mediator of the innate immune system. Poultry receiving β-glucan from *Saccharomyces cerevisiae* was shown to express down-regulated IL-8 compared to non-supplemented chicks. The down-regulation of IL-8 following β- glucan supplementation suggests that the β-glucan functions as an anti-inflammatory immunomodulatory agent [52].

Additionally, Cox et al. [52] demonstrated that similarly to mammals, avian macrophages synthesize tumor necrosis factor (TNF)-α, IL-1β, interferon (IFN)-γ, IL-2, and IL-4. When IL-4 is produced, it induces the development of type-2 helper T cells (Th2). Th2 cells primed cells are able to produce IL-5, IL-6, IL-10, and IL-13, which are cytokines involved in the humoral immune response. Avian macrophages also synthesize IL-18. IL-18 is a pro-inflammatory cytokine responsible for inducing the migration of its target cells to inflammation sites [52]. Following exposure to a pathogen, IL-18 together with IL-12 induce an overt cell-mediated immune response. Both cytokines induce priming of Th cells to secrete IFN-γ. IFN-γ plays an essential role in further activating macrophages and plays a central role in promoting Th1 cell differentiation [52]. Dietary β-glucan to day-old chicks was demonstrated to upregulate IL-18 expression in the jejunum seven days after feeding [52]. He reported that IFN-γ, was downregulated in the β-glucan supplemented chicks compared to those receiving the un-supplemented control diet.

β-glucans have been shown to induce a T helper 1 (Th1) response, suggesting that they are implicit in stimulating macrophage production and proliferation of T cells into CD8+ T cells [52]. Β-glucans have also been studied as a viable source of prebiotics, and as a potential antibiotic replacement due to the ability of gut microbiota to protect the host organism from enteric infection. T regulatory (Treg) cell percentages were increased with the supplementation of β-glucans in the diet [53]. β-glucans extracted from barley, seaweed, and mushrooms were shown to support the growth of three different *Bifidobacteria* species, indicating their efficacy in stimulating gut microbiota growth [54]. 

The use of β-glucans in animals not only limits infection occurrence but also improves animal growth and development and significantly helps in decreasing of antibiotics usage [55]. These findings led to significant use of glucans in aquaculture, where β-glucan has been found to improve survival against experimental infections and reduce stress (for review see [56]). An early glucan bath during embryo development was shown to increase the growth and size of larvae, stressing the importance of glucan in farmed animals even more [57]. Zhang et al. [58] reported that β-glucan supplemented at low concentrations in broilers significantly improved their growth rate. It was also reported that administration to broilers of only 40 ppm of yeast-derived β-glucans induce the increase in their body weight during their growth phase, and concomitantly increase the relative weight of their gizzard, thus contributing to the increase of their digestive capacity, which may account for the growth effect [59]. 

The effects of β-glucans on the relative weight of the spleen and the bursa of Fabricius varied with studies, depending on the dose and presence of pathogen challenges [60,61] and additionally yeast β-glucan was as effective in promoting growth of broiler chickens as virginiamycin, an antibiotic routinely added to broiler diets at subtherapeutic levels [60]. Various effects on several visceral organs were observed in chickens supplemented with β-glucans in feed. A study conducted by Cho et al. [27] showed that dietary supplementation of yeast cell wall (YCW) β-glucans induced a moderate weight gain of the spleen and the bursa of Fabricius in unchallenged birds. Additionally, chickens supplemented with mannoprotein and β-glucans showed enhanced thymus weight and concomitantly induced the increase of the villus height of the jejunum mucosa of chickens [62], which may raise the total surface area of nutrient absorption. 

In this regard, Zhang et al. [58] reported that supplementing poultry diet with β-glucans led to improved growth performance as measured by daily average gain and ratio of feed conversion [26,43,58,63].

Different chicken genotypes have been shown to react differently to β-glucan supplementation [64]. Gene expression of IL-4 and L-18 in a Broiler line was not affected to a three-week β-glucan supplementation regimen, but the supplementation did exert a significant effect on chickens of the Fayoumi line. This finding indicates that the immunomodulatory supplementation effect does not yield similar beneficial effects in all avian gene lines.

## 6. Glucans as Effective Measure Against Foodborne Disease Infection in Poultry 

β-glucans have been shown to exert positive effects against various enteric pathogens and bacterial and parasite infectious diseases by upregulating immune-related activities; however, its role as an antibiotic alternative in domestic birds is not much studied. β-glucans may use various mechanisms to improve the growth and performance of the poultry industry: β-glucans can participate in the intestinal clearance of the most significant pathogens such as *E. coli* and *Salmonella* spp. [34,63] via the regulation of intestinal barriers and tight proteins and stimulation of phagocytosis, thus inhibiting major pathogen invasion into organs [34]. β-glucan supplementation can enhance goblet cell number and villus height in the ileum [62], as well as restore villus damage/loss by *Salmonella* spp. challenge [65]. 

β-glucans supplementation can improve the villus height and crypt depth loss during Eimeria/coccidia infection [52,61]. β-glucans supplementation can upregulate mucin-2 production from goblet cells, modulate intestinal profile of cytokines, and reduce lesion severity and inflammation in the intestine of broilers infected with Eimeria. A recent study conducted by Omara et al [66] regarding *Eimeria* induced coccidiosis, consisting of dietary treatments with 0.1% β-glucan following *Eimeria* stimulated coccidiosis in poultry. They demonstrated that significant effects resulted from the dietary β-glucan supplementation to chicken diets as they induced a significant modulation of the immune response to the *Eimeria* challenge in various avian tissues.

β-glucans supplementation can induce increased sIgA secretion and upregulate the numbers of goblet cells that maintain the integrity of the mucous protective layers for expulsion of enteric pathogen invasion [67]. β-glucan supplementation upregulates the expression of tight junctions’ proteins such as occluding and claudin-1, leading to the upgraded elimination of *Salmonella Typhimurium* [65] and concomitantly inhibits *Salmonella* colonization of the caeca and liver, respectively [34,68]. These mechanisms associated with β-glucans relate to the improvement of the intestinal epithelial cell functions but no mechanism regarding direct killing or binding of β-glucans to enteric pathogens is available to date. 

*Salmonella* infection is one of the most common causes of foodborne disease worldwide and is one of the four key global causes of diarrheal diseases; it results in over 250,000 human deaths annually [19]. Poultry meat and eggs is a common source of Salmonella and are most commonly food sources associated with this disease [69]. Broiler chickens fed with yeast β-glucan showed a drastic reduction in the *Salmonella* counts in different internal organs [43], indicating the potential of yeast β-glucan in the prevention or reduction of food-borne *Salmonella* infections.

A serotype such as *S. Typhimurium* can be zoonotic and cause infection in humans, with poultry being the major reservoir. Infected animals express different symptoms and, in some instances, the infection may lead to the death of the poultry. It was demonstrated that in *S. typhimurium* infected poultry, the number of goblet cells decreased. Supplementation with β-glucan, however, significantly increased the number of goblet cells in the jejunum of these challenged animals [70]. This suggests that β-glucan supplementation of poultry feed play a significant role in improving their gut health during a bacterial challenge.

Intestinal secretory immunoglobulin A (sIgA) is produced in response to microbial- and food-derived antigens and plays different roles in intestinal mucosal secretions and provides a line of defense in protecting the intestinal epithelium from pathogenic microorganisms and maintaining the mucosal layer. It acts as the first line of defense against pathogens and facilitates mucus surface colonization by commensal microbiota and regulates immune homeostasis [71].

*S. typhimurium* bacteria in infected chicks have been shown to exert major cellular damage of the intestinal mucosa and induce a significant reduction in villus height and the villus height to crypt depth ratio of the jejunum [65]. However, dietary yeast β-glucan supplementation of these chicks challenged with *S. typhimurium* resulted in higher villus height and villus height to crypt depth ratio than for the un-supplemented chicks [65]. 

## 7. The Putative Mechanisms by which β-glucans may Exert Antibiotic Alternative Effects

**Figure 1:**
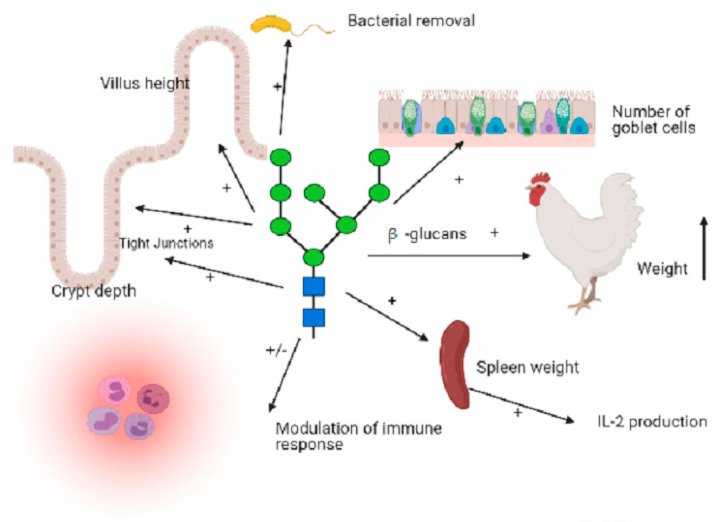
Summary of the activities exerted by β-glucans obtained from several sources. These activities include enhancement of goblet cell number and upregulated mucin-2 production, improvement of villus height and crypt depth, increased immune response by altering the cytokine profiles, induced increased expression of tight junction proteins, thus avoiding the invasion of pathogens into the deeper layers of the GI tract, significantly increased the relative weight of the spleen stimulating spleen genes causing the activation of the systemic immune function.

There are various mechanisms by which β-glucans dietary supplementation have been shown to play a role as effective antibiotic alternatives in poultry (See Figure 1):

**(I)** Dietary β-glucan supplementation to poultry can enhance goblet cell number upregulated mucin-2 production from goblet cells [62,70]. Goblet cells are cellular structures that are responsible to maintain the integrity of the mucous protective layers for effective removal of enteric pathogens.

**(II)** β-glucans supplementation to the diet of poultry improves villus height and crypt depth and their ratio in the duodenum and ileum [61].

**(III)** β-glucan supplementation has been demonstrated to modulate the intestinal profile of cytokines that activate the major histocompatibility complex, which afterwards activate monocytes and macrophages [58]. Β-glucan has also been shown to enhance immune response by altering the cytokine profiles of chickens [66]. This results in increased immune response that involves the activation of T-helper cells, cytotoxic macrophages, and natural killer cells, as well as the induction of proliferation and differentiation of T-cells [72]. It seems like IL-1, IL-2, IFN-γ, and TNF-α may act as the key signaling molecules responsible for regulating this immune network [36]. 

**(IV)** β-glucan supplementation has been demonstrated to reduce the severity of intestinal lacerations and inflammation in broilers suffering from *Eimeria/coccidia* infection [52,61]. 

**(V)** β-glucan supplementation has been demonstrated to induce the increased expression of jejunal tight junction proteins such as occludin and claudin-1 [65] avoiding the invasion of pathogens into the deeper layers of the gastrointestinal tract. 

**(VI)** β-glucan supplementation has been demonstrated to restore the villus damage/loss exerted by *Salmonella* poultry infection [65].

**(VII)** Dietary supplementation with β-glucans to poultry has been demonstrated to increase the intestinal clearance of various important pathogens such as *E. coli* and *Salmonella* spp. [34,63] via the protection of intestinal barriers, and concomitantly inducing the increased expression of jejunal tight junction proteins such as occludin and claudin-1, which ultimately led to the increased removal of these pathogens [65] alongside the inhibition of the colonization and invasion of *Salmonella* in the caeca and liver, respectively [34,68,73].

**(VIII)** Dietary supplementation with β-glucans to poultry has been demonstrated to stimulate phagocytosis, which eventually suppresses invasion of pathogen into organs [34] and thus contributes to the improved growth and performance of poultry. 

**(IX)** In chicken, the spleen serves both as a reservoir of leukocytes as well as a target organ of immune cells activation. Zhang et al. [31] demonstrated that feeding β-glucan significantly increased (3.9%) the relative weight of the spleen. Stimulation of spleen genes generally mirrors the activation of the systemic immune function [64]. This activation includes upregulation of genes to interleukins such as IL-4 and IL-18. Mushrooms derived β-glucans have been shown to promote splenocyte proliferation and interleukin-2 production in chickens [73]. 

**(X)** β-glucan supplementation was shown to increase the production of new immunocytes [74]. Additionally, β-glucan supplementation has been shown to increase the phagocytic function of macrophages [50]. Β-glucans increase the phagocytic, bactericidal killing, and oxidative burst of heterophils [34]. Cheng et al. [75] showed that β -glucan of the fungus *Schizophyllum commune* significantly increased the chemotaxis activity of macrophages.

**(XI)** β-glucans derived from the yeast *Saccharomyces cerevisiae* were demonstrated in vitro to be cytoprotective and genoprotective in chicken lymphocytes [76]. The researchers surmised that the cytoprotection resulted from the role of β-glucan in repair or blocking of mutations during the life cycle of the cell.

Table 1 summarizes the most important effects exerted by extracted glucans from several nutritional sources on poultry physiology and immune state.

## 8. Conclusions

From the information provided above, we can conclude that β-glucans may provide an efficient tool for producers trying to reduce or eliminate the use of antibiotics in poultry diets.

Therefore, β-glucans can act as a safe dietary solution to poultry growers against enteric pathogens by increasing goblet cell numbers, upregulating expression of intestinal TJ proteins, and modulation of cytokines. These effects allow β-glucans to be considered as a growth promoter in broilers. As a growth promoter and antibiotic alternative, further studies are required to optimize the dosage and source of β-glucans to determine its effects on growth performance and mechanisms against enteric pathogens. In conclusion, β-glucans may be used as a growth promoter and antibiotic alternative.

## Figures and Tables

**Table 1:** Most important effects of β-glucan in chicken.

Glucan	Effects	Reference
Yeast	Increased phagocytosis, microbicidal killing	[34]
Yeast	Improved growth parameters	[37]
Yeast	Inhibition of immunosuppression	[38]
Yeast	Improved efficacy of vaccines	[39]
Yeast	Improved GI health	[43]
Yeast	Increased phagocytosis	[50]
Yeast	Increased phagocytosis, IL-2 production, growth, decreased stress	[77]
Yeast	Upregulation of iNOS intestinal expression, downregulation of IL-8	[52]
Yeast	Increased numbers of Treg cells	[53]
Yeast	Improved growth	[58]
Yeast	Increased anti-infection response	[43]
Yeast	Improved intestinal mucosa	[65]
Yeast	Increased chemotaxis	[75]
Barley	Increased intestinal viscosity	[42]
Mushroom	Increased phagocytosis, microbicidal killing	[78]
Mushroom	Increased proliferation of splenocytes, IL-2 production	[73]
